# The Evolutionarily Conserved E3 Ubiquitin Ligase AtCHIP Contributes to Plant Immunity

**DOI:** 10.3389/fpls.2016.00309

**Published:** 2016-03-15

**Authors:** Charles Copeland, Kevin Ao, Yan Huang, Meixuizi Tong, Xin Li

**Affiliations:** ^1^Michael Smith Laboratories, University of British ColumbiaVancouver, BC, Canada; ^2^Department of Botany, University of British ColumbiaVancouver, BC, Canada

**Keywords:** ChIP, E3 lligase, plant immunity, chaperones, SGT1, HSP90 heat-shock proteins

## Abstract

Plants possess a sophisticated immune system to recognize and respond to microbial threats in their environment. The level of immune signaling must be tightly regulated so that immune responses can be quickly activated in the presence of pathogens, while avoiding autoimmunity. HSP90s, along with their diverse array of co-chaperones, forms chaperone complexes that have been shown to play both positive and negative roles in regulating the accumulation of immune receptors and regulators. In this study, we examined the role of AtCHIP, an evolutionarily conserved E3 ligase that was known to interact with chaperones including HSP90s in multicellular organisms including fruit fly, *Caenorhabditis elegans*, plants and human. *Atchip* knockout mutants display enhanced disease susceptibility to a virulent oomycete pathogen, and overexpression of *AtCHIP* causes enhanced disease resistance at low temperature. Although CHIP was reported to target HSP90 for ubiquitination and degradation, accumulation of HSP90.3 was not affected in *Atchip* plants. In addition, protein accumulation of nucleotide-binding, leucine-rich repeat domain immune receptor (NLR) SNC1 is not altered in *Atchip* mutant. Thus, while AtCHIP plays a role in immunity, it does not seem to regulate the turnover of HSP90 or SNC1. Further investigation is needed in order to determine the exact mechanism behind AtCHIP’s role in regulating plant immune responses.

## Background

### Plant Immunity

Plants have evolved sophisticated immune systems to recognize and defend against infections by diverse microbial pathogens (Dangl et al., 2013). Receptor-like kinases on the cell surface can recognize conserved microbial features termed pathogen associated molecular patterns (PAMPs) from microbes, and trigger a relatively weak PAMP-triggered immunity (PTI) response (Macho and Zipfel, 2014). While PTI is effective at preventing infection by many microbes, successful pathogens are able to deliver effector molecules into the plant cell to suppress PTI and promote virulence (Dangl et al., 2013). An additional layer of the plant immune system involves resistance (R) proteins, which can recognize specific pathogen effectors and trigger a more rapid and robust effector-triggered immunity (ETI).

Plant genomes contain a large number of *R* genes, most encoding proteins with nucleotide-binding, leucine-rich repeat domains (NLRs; or Nod-like receptors). Typical NLRs in plants contain Toll-interleukin-1 receptor (TIR) or coiled-coil (CC) domains at their N termini (Jones and Dangl, 2006; Li et al., 2015). Immune responses mediated by TIR-type NLR (TNL) proteins often require the immune signaling module EDS1/PAD4/SAG101, but the detailed molecular events surrounding NLR activation are largely unclear (Wiermer et al., 2005). Rapid and strong ETI induction is important for preventing pathogen infections (Jones and Dangl, 2006). However, ETI signaling must be suppressed in healthy wild-type plants, as mutants with constitutive ETI can be dwarfed and often show spontaneous cell death (Li et al., 2001; Yang et al., 2010). For example, a point mutation in Suppressor of *npr1*, constitutive 1 (SNC1), a TNL protein in *Arabidopsis thaliana*, results in the autoimmune *snc1* mutant, which exhibits dwarfism, curled-leaf morphology, and enhanced resistance against virulent pathogens (Li et al., 2001; Zhang et al., 2003). More recent studies have shown that SNC1 and other NLRs are regulated post translationally, through degradation by the 26S proteasome pathway (Cheng et al., 2011; Huang et al., 2014b). E3 ubiquitin ligase complex containing the F-box protein CPR1 targets SNC1 for ubiquitination and subsequent degradation (Cheng et al., 2011). The point mutation in *snc1* plants stabilizes the snc1 protein, increasing its steady state level and resulting in the autoimmune phenotype (Cheng et al., 2011). The sensitized background of *snc1* has been used as an efficient tool to elucidate further components of plant immunity, as it can be used to search for both enhancers and suppressors.

### HSP90-Containing Chaperone Complexes Play Positive and Negative Roles in Immunity

Chaperone complexes containing HSP90s play important roles in ensuring R protein stability and function (Kadota and Shirasu, 2012). The contribution of HSP90s is not straightforward, as mutations in different isoforms or different alleles of the same isoform often have differing, or even opposite phenotypic effects. For example, mutations in *HPS90.2* leads to reduced *RPM1* levels and function (Hubert et al., 2003; Takahashi et al., 2003). However, different alleles of *hsp90.2* and *hsp90.3* can result in increased NLR stability leading to autoimmunity, such as with SNC1 and RPS2 (Huang et al., 2014a). This could be explained by HSP90’s differential chaperone roles in different protein complexes. They could serve in NLR activation complex, and at the same time, be involved in the NLR degradation complex as SCF (Skp1, Cullin 1 and F-box) E3 ligase complex members.

Adding to the complexity of HSP90’s role in NLR function are the many co-chaperones that function with HSP90, which are also presumably crucial for proper NLR folding and function. Mutations in *SGT1b*, or alleles of *HSP90* that abolish the interaction between SGT1b and HSP90, cause a reduction in R protein levels (Hubert et al., 2003; Zhang et al., 2008; Shirasu, 2009; Kadota and Shirasu, 2012). Mutations in *SGT1b* and *RAR1* both partially compromise resistance mediated by RPP5, but these phenotypes are additive, indicating that they likely function independently in RPP5 signaling (Austin et al., 2002). The interaction between HSP90 and its co-chaperones must be important, as specific alleles of *hsp90.2* can partially restore NLR signaling that is abolished in *rar1* mutants (Hubert et al., 2003).

Like HSP90s, in addition to SGT1’s positive roles in plant immune receptor stability and activation, SGT1 also serves in SCF-mediated NLR protein turnover. SGT1 interacts directly with Skp1, a common component of SCF E3 ligase complexes (Kitagawa et al., 1999). In *Arabidopsis*, mutations in *SGT1b* and *SRFR1*, encoding an interactor of SGT1, lead to higher accumulation of NLR proteins RPS5, SNC1, and RPS2 (Holt et al., 2005; Li et al., 2010). These diverse roles of SGT1 mirror those of HSP90s.

### CHIP is a Conserved E3 Ligase that Interacts with HSP Chaperones

**FIGURE 1:**
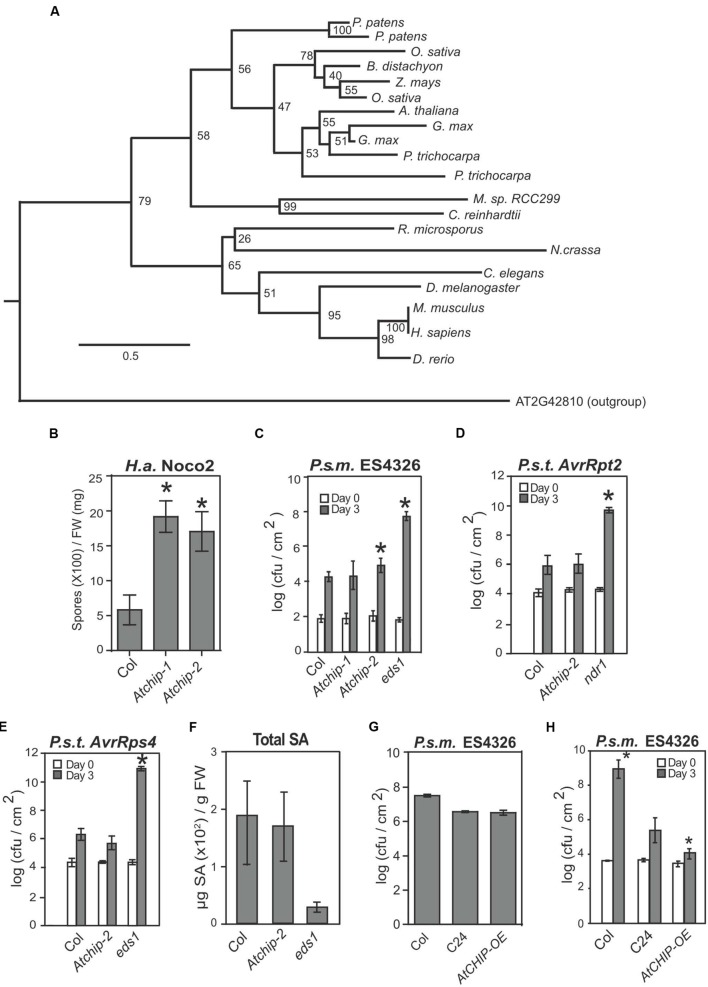
***Atchip* knockout mutants exhibit enhanced susceptibility to virulent but not avirulent pathogens, while overexpression of *AtCHIP* leads to enhanced resistance only at lower temperature. (A)** Maximum-likelihood tree of CHIP sequences from representative eukaryotes. Node labels represent bootstrap values from 1000 replicates. The scale bar represents the average number of substitutions per site in each branch. Organisms shown in the tree are *Arabidopsis thaliana*, *Brachypodium distachyon, Caenorhabditis elegans, Chlamydomonas reinhardtii, Danio rerio, Drosophila melanogaster, Glycine max, Homo sapiens, Micromonas sp.* RCC299, *Mus musculus, Neurospora crassa*, *Oryza sativa, Physcomitrella patens, Populus trichocarpa, Rhizopus microspores*, and *Zea mays*. **(B)** Growth of *Hyaloperonospora arabidopsidis* Noco2 on Col, *Atchip-1*, and *Atchip-2* plants. Two-week-old seedlings were sprayed with a spore suspension at a concentration of 5 × 10^4^ spores per mL, and oomycete spores grown on leaf surface were quantified 7 days later using a hemocytometer. Asterisks indicate a significant difference from Col (*p* < 0.05) as determined by *t*-tests. The experiment was repeated more than three times with similar results. **(C)** Growth of *Pseudomonas syringae* pv *maculicola* ES4326 on wild type Col, *Atchip-1*, *Atchip-2*, and *eds1* plants (*eds1* serves as a susceptibility control). Leaves of 4-week-old plants were infiltrated with a bacterial suspension in 10 mM MgCl_2_ at *OD*_600_ = 0.0001. Leaf disks within the infected area were taken immediately after infiltration (Day 0) and 3 days after infiltration (Day 3) to quantify bacterial colony-forming units (cfu). Bars represent mean values of three (Day 0) or five (Day 3) replicates ± SD. Asterisks indicate a significant difference from Col (*p* < 0.05) as determined by *t*-tests. **(D)** and **(E)** Growth of *P. syringae* pv tomato DC3000 expressing *AvrRpt2* **(D)** or *AvrRps4* **(E)** on wild type Col, *Atchip-2*, and *ndr1* or *eds1* plants. Leaves of 4-week-old plants were infiltrated with a bacterial suspension in 10 mM MgCl_2_ at *OD*_600_ = 0.001. Leaf disks within the infected area were taken immediately after infiltration (Day 0) and 3 days after infiltration (Day 3) to quantify bacterial colony-forming units (cfu). Bars represent mean values of three (Day 0) or five (Day 3) replicates ± SE. Asterisks indicate a significant difference from Col (*p* < 0.05) as determined by ANOVA followed by Tukey’s HSD test. **(F)** Salicylic acid (SA) accumulation in Col, *Atchip-2*, and *eds1* induced with *P. syringae* pv tomato DC3000 carrying *AvrRps4*. Plants were infiltrated with bacterial suspension in 10 mM MgCl_2_ at *OD*_600_ = 0.2. Tissue was harvested after 24 h for total SA extraction and quantification using an HPLC. Asterisks indicate a significant difference from Col (*p* < 0.05) as determined by ANOVA followed by Tukey’s HSD test. **(G)** Growth of *P. syringae* pv. *maculicola* ES4326 on wild type Col, C24, and *AtCHIP-OE* plants. Leaves of 4-week-old plants were infiltrated with a bacterial suspension in 10 mM MgCl_2_ at *OD*_600_ = 0.001. Leaf disks within the infected area were taken 3 days after infiltration (Day 3) to quantify bacterial colony-forming units (cfu). Bars represent mean values of five (Day 3) replicates ± SE. Asterisks indicate a significant difference from C24 as determined by ANOVA followed by Tukey’s HSD test. **(H)** Growth of *P. syringae* pv. *maculicola* ES4326 on wild-type Col, C24, and *AtCHIP-OE* plants under low temperature. Plants were transferred to 18°C for at least 1 week, and infiltrated as in **(G)**. Asterisks indicate significant difference (*p* < 0.05) from C24, as determined by a one-way ANOVA followed by Tukey’s HSD test.

Given the importance of chaperone complexes in both positive and negative regulation of NLR accumulation, it is likely that other chaperone-interacting proteins also play roles in regulating NLRs. One evolutionarily conserved candidate protein with well-characterized interactions with HSP90 and HSP70 in animals is C-terminus of Hsc70 Interacting Protein (CHIP; AtCHIP in *Arabidopsis*), an E3 ligase that ubiquitinates unfolded client proteins bound by the chaperone complexes (Connell et al., 2001; Murata et al., 2003). CHIP-encoding genes are broadly found in eukaryotes including fungi, plants, and animals (**Figure 1A**), mostly as single-copy genes. Intriguingly, no CHIP homolog was identified in budding or fission yeast. First identified as an HSP-interacting protein, CHIP is a unique E3 ligase that links heat-shock chaperone complexes with ubiquitination and proteosomal degradation, generally of misfolded substrate proteins (Murata et al., 2003). Work by Qian et al. (2006) suggests that the target of the E3 ligase activity depends on the amount of unfolded client proteins present, where CHIP preferentially ubiquitinates chaperone-bound substrates before ubiquitinating the chaperones themselves. Interestingly, CHIP does not seem to target a specific substrate protein like most other E3s, but rather, relies on the selectivity of its associated chaperones for client substrates. The crystal structure of mammalian CHIP, both alone and in interaction with the chaperone HSC70, has been solved (Zhang et al., 2005, 2015). CHIP protein contains a C-terminal U-box domain and an N-terminal tetratricopeptide repeat (TPR). Forms of CHIP with mutations in the TPR region retain self-ubiquitination activity, indicating that the U-box domain is sufficient for E3 ligase activity (Zhang et al., 2015). However, the TPR domain is required for the interaction with Hsc70 and ubiquitination of misfolded client proteins, emphasizing the importance of protein–protein interactions for CHIP function.

Previous studies on AtCHIP in *Arabidopsis* have identified a role for AtCHIP in response to abiotic stress. Yan et al. (2003) confirmed the E3 ligase activity of AtCHIP, and found that the expression of *AtCHIP* is up-regulated by osmotic and temperature stresses; however, constitutive overexpression of *AtCHIP* results in increased susceptibility to heat and chilling. Like animal CHIP, AtCHIP also interacts with HSC70, facilitating the degradation of plastid-targeted precursor proteins, preventing them from building up in the cytosol (Lee et al., 2009). In addition, AtCHIP was shown to interact with, ubiquitinate, and reduce the cellular levels of chloroplast proteins Clp4, a subunit of the chloroplast Clp proteolytic complex, and FtsH1/2, two subunits of the chloroplast Fts protease complex (Shen et al., 2007a,b). These results indicate a role for AtCHIP in degradation of multiple protein targets through the 26S proteasome pathway. Additionally, AtCHIP interacts with and ubiquitinates PP2A (protein phosphatase 2A), which is involved in the response to low-temperature. However, overexpression of *AtCHIP* does not affect the steady-state levels of PP2A isoforms, and PP2A activity is increased in *AtCHIP-OE* plants under low-temperature conditions, indicating that ubiquitination by AtCHIP may play regulatory roles beyond proteasomal degradation (Luo et al., 2006).

### AtCHIP and Plant Immunity

The function of AtCHIP in immune signaling is largely unexplored. Given that animal CHIP interacts with HSP90s and HSP70, AtCHIP interacts with HSC70-4 in *Arabidopsis*, and HSP90 is involved in the stability regulation of a number of NLR proteins, we hypothesized that AtCHIP might play a role in regulating NLRs during plant immune responses. According to publically available microarray expression data found on *Arabidopsis* eFP browser, *AtCHIP* expression is indeed induced by infections with virulent *Pseudomonas syringae* pv. *maculicola* (*P.s.m.*) ES4326 or the same strain carrying the effector AvrRpt2, as well as by treatment with SA (**Supplementary Figure S1**). We therefore investigated the potential roles of AtCHIP in plant immunity, in order to expand our perspectives on its function in plants. Using a reverse genetics approach, we examined the immune phenotypes of *Atchip* loss-of-function mutants, as well as plants overexpressing *AtCHIP* (*AtCHIP-OE)*.

## Experimental Results

### *Atchip* Mutants Show Increased Susceptibility to Virulent But Not Avirulent Pathogens

In order to determine whether AtCHIP plays a role in immune regulation, we first obtained two exonic T-DNA knockout alleles of *Atchip*, named *Atchip-1* (SALK_048371) and *Atchip-2* (SALK_059253), and examined their immune phenotypes against a variety of pathogens. *Atchip* plants of both mutant alleles exhibit enhanced disease susceptibility against the oomycete pathogen *Hyaloperonospora arabidopsidis* (*H.a.*) Noco2 (**Figure 1B**). We also observed slight enhanced susceptibility against the virulent bacterial pathogen *P. syringae* pv. *maculicola* (*P.s.m.*) ES4326, although this was not always significant (**Figure 1C**). In addition, *Atchip-2* plants showed wild-type levels of resistance to *P. syringae* pv. *tomato* (*P.s.t.*) DC3000 strains carrying either *AvrRpt2* or *AvrRps4*, which are avirulent on the Columbia (Col) ecotype due to the presence of the NLRs *RPS2* and *RPS4*, respectively (**Figures 1D,E**). Furthermore, when the plants were infiltrated with a high dose of *P.s.t.* DC3000 *AvrRps4*, the defense hormone salicylic acid (SA) accumulated to the same level as wild-type (**Figure 1F**). Therefore, *Atchip* positively contributes to basal immunity, but does not appear to affect NLR-mediated immunity or SA accumulation.

### Overexpression of *AtCHIP* Causes Enhanced Disease Resistance under Low Temperature Conditions

Because *Atchip* knockout mutants exhibit a mild enhanced disease susceptibility phenotype, we hypothesized that increased levels of AtCHIP might cause enhanced disease resistance. We obtained plants overexpressing *AtCHIP* (*AtCHIP-OE*), which were generated in the C24 ecotype background and described previously by Yan et al. (2003). Under normal growth conditions, *AtCHIP-OE* plants supported similar *P.s.m.* ES4326 growth as the C24 controls (**Figure 1G**). Note that C24 is known to exhibit enhanced resistance to virulent pathogens (Lapin et al., 2012).

While *AtCHIP-OE* was previously reported to show wild-type-like morphology under standard growth conditions, they are temperature sensitive, as exposure to 7°C results in severely stunted growth and electrolyte leakage (Yan et al., 2003), indicative of autoimmunity. Therefore, we predicted that overexpression of *AtCHIP* may result in enhanced disease resistance under low temperature conditions. When plants were grown at 20°C, and transferred to 18°C for at least 1 week before infection, we observed a significant reduction in bacterial growth following *P.s.m.* ES4326 infection (**Figure 1H**). Thus, consistent with the enhanced susceptibility in *Atchip* knockout mutants, overexpression of *AtCHIP* causes enhanced disease resistance, supporting a positive role AtCHIP plays in immune regulation.

### The Autoimmune Phenotype of *snc1* Mutants Does Not Depend on *AtCHIP*

**FIGURE 2:**
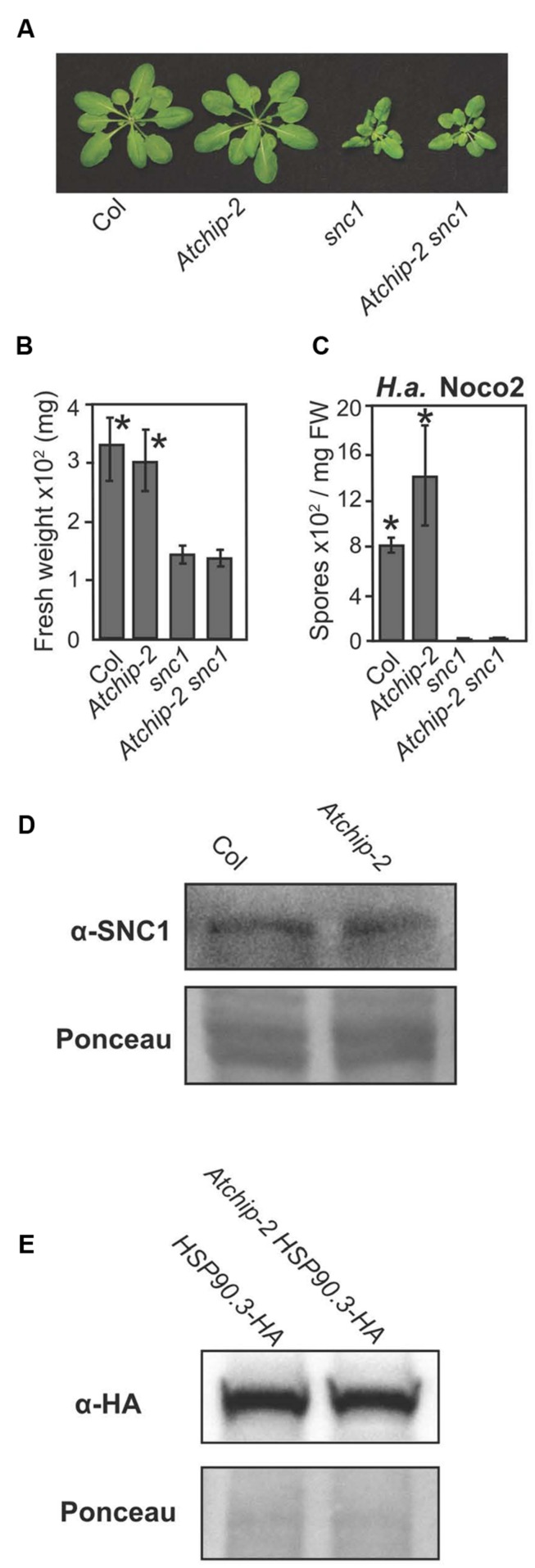
***Atchip-2* knockout does not suppress the *snc1* phenotype, and SNC1 and HSP90 levels are not altered in *Atchip-2* plants. (A)** Morphology of 4-week-old soil-grown plants of the indicated genotypes. **(B)** Fresh weights of plants of the indicated genotypes. Asterisks indicate significant differences from *snc1* at *p* < 0.05, as determined by one-way ANOVA and Tukey’s HSD test. **(C)** Resistance against *H.a.* Noco2 in Col, *Atchip*, *snc1*, and *snc1 Atchip-2* plants. Two-week-old seedlings were sprayed with a spore suspension at a concentration of 10^5^ spores per mL, and spores were quantified 7 days later using a hemocytometer. Asterisks indicate a significant difference (*p* < 0.05) from *snc1*, as determined by *t*-tests. **(D)** SNC1 protein levels in *Atchip-2* plants. Total protein from 3-week-old plants of Col and *Atchip* genotypes was subjected to immunoblotting with an α-SNC1 antibody (Li et al., 2010). Ponceau staining is shown as a loading control. **(E)** HSP90.3-HA levels in Col and *Atchip-2* genotypes. Total protein was extracted from the aerial tissue of 2-week-old seedlings of the indicated genotypes. HSP90.3-HA levels were examined using immunoblotting with an α-HA antibody. Ponceau staining is shown as loading control.

The *Atchip* mutation alone causes only mild enhanced disease susceptibility. However, single mutants of many genes involved in NLR regulation do not show strong enhanced disease susceptibility, yet can dramatically suppress the autoimmune phenotypes of *snc1*, an autoimmune mutant carrying a gain-of-function mutation in a TNL (Li et al., 2001; Johnson et al., 2013). We therefore created a *Atchip-2 snc1* double mutant to test whether the *Atchip* mutation could suppress the autoimmune phenotypes of *snc1*. The *Atchip-2 snc1* double mutants displayed the same dwarf, curled-leaf morphology as *snc1* and were not significantly larger than *snc1* single mutant as examined by plant fresh weight (**Figures 2A,B**). *H.a.* Noco2 infection further confirmed that the *Atchip-2 snc1* plants retain the enhanced disease resistance of *snc1* (**Figure 2C**). Therefore AtCHIP does not seem to contribute to SNC1-mediated immunity.

### AtCHIP Does Not Affect SNC1 Turnover

Since animal CHIPs were reported to associate with evolutionarily conserved chaperones SGT1 and HSP90s, which contribute to NLR SNC1 turnover in *Arabidopsis*, we examined whether AtCHIP is also involved in NLR turnover (Connell et al., 2001; Zhang et al., 2008). When we examined the SNC1 protein levels in the *Atchip-2* backgrounds; however, no obvious alteration in SNC1 levels was observed compared to the wild-type controls (**Figure 2D**). These results are consistent with the inability of *Atchip* to suppress the autoimmune phenotype of *snc1.*

### HSP90.3-HA Levels Are Not Affected in *Atchip* Knockout Plants

Expression of *CHIP* in human cell lines reduces the accumulation of HSP70 when the level of unfolded clients is low (Qian et al., 2006). Human CHIP protein can also ubiquitinate chaperones HSP90 and HSP70 *in vitro*, creating ubiquitin chains that contain K48 linkages, which is predicted to mark the HSPs for degradation (Kundrat and Regan, 2010). These findings led to the hypothesis that AtCHIP may play a role in the regulation of HSP90 levels. To test this, a *HSP90.3-HA* transgene was introduced into the *Atchip-2* background by crossing (Huang et al., 2014a). However, no difference in the HSP90.3-HA protein levels was observed in the *Atchip-2* background compared to the wild-type (**Figure 2E**). Therefore AtCHIP is unlikely to target HSP90s for ubiquitination and degradation.

## Current Perspectives and Future Directions

Animal CHIPs have been shown to interact with HSP chaperones, and in some cases modulate their turnover. Chaperones and their interactors play both positive and negative roles in immune signaling. For example, the stability of many NLR proteins is dependent on chaperone HSP90 and co-chaperones SGT1 and RAR1 (Kadota and Shirasu, 2012; Li et al., 2015). Here, we examined whether CHIP, as a component of animal chaperone complexes, similarly contributes positively or negatively to plant immune signaling. We observed a slight enhanced disease susceptibility in *Atchip* mutants and enhanced resistance in *AtCHIP* overexpression lines (**Figure 1**). However, we did not observe defects in ETI mediated by specific NLR proteins in *Atchip* mutant plants. *Atchip* mutants retain resistance to avirulent *P.s.t.* DC3000 expressing *AvrRps4* and *AvrRpt2* (**Figures 1D,E**), and *Atchip snc1* double mutants displayed the same level of autoimmunity as *snc1* alone (**Figures 2A–C**). SNC1 protein levels were also unaffected in the *Atchip* knockout (**Figure 2D**). The lack of ETI phenotypes in *Atchip* mutant argues against its predicted roles in chaperone-assisted NLR functions. However, as *Atchip* does exhibit slight susceptibility against a virulent oomycete pathogen (**Figure 1**), it must contribute to plant immune regulation through a yet-to-be-identified mechanism. One alternative explanation could be that AtCHIP is perhaps only involved in the regulation of other untested NLRs besides SNC1, RPS2, or RPS4.

Multiple studies have shown that CHIP can ubiquitinate the chaperones HSP70 and HSP90, which often results in a reduction in the chaperone levels (Qian et al., 2006; Kundrat and Regan, 2010). While we found no difference in the levels of HSP90.3-HA in *Atchip* mutant compared to wild-type, we cannot rule out that AtCHIP may play a role in immunity by affecting HSP90 function in some other way. Overexpression of CHIP does not reduce the stability of HSP70 and HSP90 in all cases, and ubiquitination of these chaperones may have other roles (Jiang et al., 2001; Morales and Perdew, 2007; Zhou et al., 2014; Edkins, 2015). Additionally, CHIP may affect the function of chaperone complexes by competing for protein–protein interaction sites on HPS90s. For example, the co-chaperone Hop facilitates the transfer of client proteins from HSP70 to HSP90, by simultaneously binding to both complexes (Edkins, 2015). However, binding of CHIP to HSP90 reduces the ability of Hop to bind to HSP90, interfering with the protein transfer and reducing the activity of HSP90 on these clients. CHIP may also target other proteins in the complex for degradation.

Typically, E3 ligases are thought to provide specificity to the ubiquitin-proteasome system, by targeting one or a small number of similar proteins (Deshaies and Joazeiro, 2009). However, CHIP appears to be unusual for an E3 ligase, as it has been shown to ubiquitinate many different substrate proteins and target them for degradation (Murata et al., 2003). While majority of CHIP’s known substrates have been identified in animal systems, the role of plant AtCHIP for regulating protein accumulation both under steady state and in response to heat stress suggests that AtCHIP has a similar function as animal CHIP (Murata et al., 2001; Zhou et al., 2014; Tillmann et al., 2015). The promiscuity of AtCHIP makes it difficult to identify the molecular mechanism underpinning its role in immunity. It is possible that one of the targets of AtCHIP is a negative regulator of immunity, and that this protein accumulates in *Atchip* plants, causing the enhanced disease susceptibility. However, it is equally plausible that loss of *AtCHIP* function causes an abnormal increase of many proteins, which together contribute to a cellular environment that reduces immune signaling.

In summary, here we provide a new perspective on the potential regulation of HSP90 and NLRs through evolutionarily conserved AtCHIP. Additional investigation is needed to elucidate the function of AtCHIP in immunity. Because mutations in HSP complexes differentially affect signaling mediated by different NLRs, experiments need to be completed to test whether *Atchip* mutants show defects in resistance from other untested NLRs. Greater insights into the dynamics of HSP90 complexes or detailed proteomic studies using *Atchip* mutants or overexpression lines may be required to identify the exact mechanism by which AtCHIP contributes to plant immunity.

## Materials and Methods

### Plant Material

Soil-grown *A. thaliana* plants were maintained in a growth chamber under 18 h light/6h dark, 22°C growth conditions. Plate-grown *A. thaliana* seedlings were grown on ½ strength MS medium in sealed plates.

### Phylogenetic Analysis

The deduced amino acid sequence of AtCHIP was used as a query in BLAST searches to identify related sequences in model eukaryotic organisms and crops. Fungal and animal sequences are from the NCBI Protein Database, and plant and algal sequences are from the PLAZA comparative plant genomics database (Van Bel et al., 2012). Mesquite was used to align sequences with MUSCLE, along with the sequence of AT2G42810, which served as an outgroup. A maximum-likelihood tree was constructed with RaxML, using the JTT model with 1000 bootstrap replicates.

### Pathogen Infections

The methods used for infection experiments involving *H.a.* Noco2 and *P. syringae* were previously described (Li et al., 2001). For *H.a.* Noco2 infections, 2-week-old seedlings were sprayed with a conidiophore suspension at a concentration noted in the figure legends. Inoculated plants were kept at 18°C at 80% humidity under a 12 h light/12 h dark cycle. The level of infection was quantified after 7 days by counting the number of condiophores present per gram of tissue using a hemocytometer. For *P.s.t.* DC3000 infections, plants were grown at 22°under a 12 h light/12 h dark cycle. Bacteria grown in LB and diluted to the indicated concentrations with 10 mM MgCl2, was used to infiltrate leaves of 4-week-old plants. Leaf disks of the infected area were taken at 0 and 3 days after infiltration to quantify bacterial colony-forming units (cfu) on agar plates with proper antibiotic selection. For low temperature treatment, plants were transferred to a chamber at 18°C under a 12 h light/12 h dark cycle for at least 1 week prior to infiltration.

### Protein Level Analysis

Total protein was extracted as in (Huang et al., 2014b). Briefly, finely ground plant tissue was homogenized in extraction buffer and centrifuged, and the supernatant was transferred to new tubes containing loading buffer. Protein was separated using SDS-PAGE and transferred to nitrocellulose membranes for immunoblotting with specific antibodies.

## Author Contributions

KA and CC carried out most of the experiments described. MT and YH isolated *Atchip-2* mutant and carried out the initial characterization. KA, CC, and XL wrote the paper. All authors read and revised the manuscript. Natural Sciences and Engineering Research Council of Canada (NSERC) Discovery Grant program funded this research.

## Conflict of Interest Statement

The authors declare that the research was conducted in the absence of any commercial or financial relationships that could be construed as a potential conflict of interest.

## Supplementary Material

FIGURE S1 ***AtCHIP* expression is induced by pathogens and SA. (A)** *AtCHIP* expression after mock treatment, or infiltration with the indicated strains of *P.s.m.*, at the indicated time points. **(B)** *AtCHIP* expression 3 h after mock treatment or treatment with 10μM SA. Data for **(A)** and **(B)** were taken from AtGenExpress.
