# Supplementary material for: The Evolutionarily Conserved E3 Ubiquitin Ligase AtCHIP Contributes to Plant Immunity
Source: Front Plant Sci. 2016 Mar 15;7:309. doi: 10.3389/fpls.2016.00309 (PMC4791365; doi:10.3389/fpls.2016.00309)
Supplement: FIGURE S1 — AtCHIP expression is induced by pathogens and SA. (A) AtCHIP expression after mock treatment, or infiltration with the indicated strains of P.s.m., at the indicated time points. (B) AtCHIP expression 3 h after mock treatment or treatment with 10μM SA. Data for (A) and (B) were taken from AtGenExpress. [file Image_1.PDF]

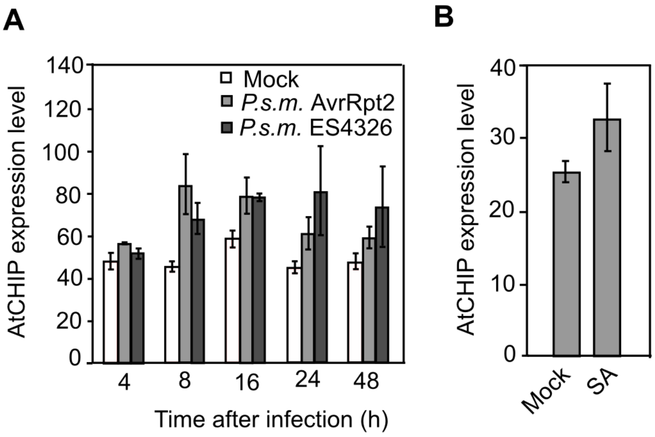

Figure S1 | *AtCHIP* expression is induced by pathogens and SA.(A) *AtCHIP* expression after mock treatment, or infiltration with the indicated strains of *P.s.m.*, at the indicated time points. **(B)** *AtCHIP* expression 3h after mock treatment or treatment with 10μM SA. Data for (A) and (B) were taken from AtGenExpress.
